# Ac2-26 reduced the liver injury after cardiopulmonary bypass in rats via AKT1/GSK3β/eNOS pathway

**DOI:** 10.1186/s13019-024-02801-z

**Published:** 2024-06-01

**Authors:** Xi-chun Xing, Zi-ying Liu, Qing Yang, Bao-wei Jia, Lin Qiu, Lu-lu Zhang, Wei Gao

**Affiliations:** 1 Department of Anesthesiology, The Second Affiliated Hospital of Harbin Medical University, 246Xuefu Road, Harbin, 150081 Heilongjiang China; 2 Department of Critical Care Medicine, Harbin Medical University Cancer Hospital, Harbin, China; 3 Department of Cardiovascular Medicine, the Second Affiliated Hospital of Harbin Medical University, Harbin, China

**Keywords:** Ac2-26, Liver injury, Cardiopulmonary bypass, Rats

## Abstract

**Objective:**

About 10% of patients after cardiopulmonary bypass (CPB) would undergo acute liver injury, which aggravated the mortality of patients. Ac2-26 has been demonstrated to ameliorate organic injury by inhibiting inflammation. The present study aims to evaluate the effect and mechanism of Ac2-26 on acute liver injury after CPB.

**Methods:**

A total of 32 SD rats were randomized into sham, CPB, Ac, and Ac/AKT1 groups. The rats only received anesthesia, and rats in other groups received CPB. The rats in Ac/AKT1 were pre-injected with the shRNA to interfere with the expression of AKT1. The rats in CPB were injected with saline, and rats in Ac and Ac/AKT1 groups were injected with Ac2-26. After 12 h of CPB, all the rats were sacrificed and the peripheral blood and liver samples were collected to analyze. The inflammatory factors in serum and liver were detected. The liver function was tested, and the pathological injury of liver tissue was evaluated.

**Results:**

Compared with the sham group, the inflammatory factors, liver function, and pathological injury were worsened after CPB. Compared with the CPB group, the Ac2-26 significantly decreased the pro-inflammatory factors and increased the anti-inflammatory factor, improved liver function, and ameliorated the pathological injury. All the therapeutic effects of Ac2-26 were notably attenuated by the shRNA of AKT1. The Ac2-26 increased the GSK3β and eNOS, and this promotion was inhibited by the shRNA.

**Conclusion:**

The Ac2-26 significantly treated the liver injury, inhibited inflammation, and improved liver function. The effect of Ac2-26 on liver injury induced by CPB was partly associated with the promotion of AKT1/GSK3β/eNOS.

## Key points

Question: The effect and mechanism of Ac2-26 on acute liver injury following CPB were unknown.

Findings: The Ac2-26 significantly ameliorated the liver injury, inhibited the inflammation, and improved liver function.

Meaning: This research furthered our understanding of the therapeutic effect of Ac2-26 on liver injury induced by CPB was positively associated with AKT1.

## Introduction

There is an increasing number of patients undergoing cardiac surgery who need the support of cardiopulmonary bypass (CPB). The incidence of liver failure postcardiac surgery is 4%, but about 10% of patients who received CPB had been diagnosed with acute liver injury [1]. The liver injury in high-risk patients could influence the outcome and mortality of patients after CPB. Currently, the major pathological mechanism of acute liver injury after CPB includes systemic inflammatory response syndrome (SIRS) and oxidative stress [2, 3]. During CPB, exposure of blood to the artificial circuits could lead to severe systemic inflammation response and release lots of inflammatory factors, which damage the endothelium, and the injured endothelial will promote the migration and infiltration of inflammatory cells into the liver [4]. Moreover, during CPB, both the hypotension and activated inflammation resulted in severe oxidative stress response [2, 5].

Ac2-26 is an active biological peptide of Annexin A1, which mainly introduced the anti-inflammatory effect of glucocorticoids. Ac2-26 can inhibit the activation of T cells with expression of CD25 and CD69 [6], and regulate the balance of Th1 and Th2 with inhibition of T-cell receptors [7, 8]. Ac2-26 also reduces the migration and infiltration of neutrophils and monocytes by formyl peptide receptors [9, 10]. We also found that Ac2-26 reduced lung and brain reperfusion injury [11, 12]. Therefore, we hypothesized that Ac2-26 can ameliorate liver injury after CPB. In this study, we performed a CPB model in rats and injected the Ac2-26 to observe the effect of Ac2-26 on liver injury after CPB. Moreover, considering the key role of AKT1 in inflammation and cell survival, we administrated the AKT1 interference RNA to investigate the mechanism of Ac2-26 in liver injury.

## Material and methods

This study was approved by the ethics committee of Harbin Medical University. All the rats and procedures were based on “The Principles of Laboratory Animal Care” and “The Guide for the Care and Use of Laboratory Animals” formulated by Harbin Medical University.

### shRNA construction

According to the sequences of AKT1 (Gen bank accession number NM 033230), the shRNA for AKT1 was designed and constructed by Invitrogen. In my preliminary study, we found that 1 × 10^5^ ifu/L AKT1 expression lentiviral solution at 15 μL/kg body weight [13] injected for 72 h significantly reduced the AKT1 protein expression. The effect of shRNA on AKT1, AKT2, and AKT3 was detected using RT-PCR.

### Animal study

Thirty-two male SD rats were randomized into sham, CPB, Ac, and Ac/AKT1 groups. The rats in the Ac/AKT1 group were injected with the shRNA to interfere with the expression of AKT1 preoperative 72 h according to the results of our preliminary study. After 72 h of injection of shRNA, these 8 rats received CPB.

The rats in the sham group only received anesthesia and cannulation. The rats in CPB, Ac, and Ac/AKT1 groups received the standard CPB for 60 min [14]. The rats in the CPB group received intravenous injections of saline, and rats in the Ac and Ac/AKT1 groups received intravenous injections of Ac2-26. All the rats were intubated with a 16-gauge cannula for mechanical ventilation. The respiratory parameters were set as Vt 8 ml/kg, respiratory rate 50/min, and fraction inspiratory oxygen 50%. The anesthesia was maintained with 2% isoflurane throughout the experiment. The CPB procedure was referred from Hirao’s study [15]. Briefly, the right femoral artery was cannulated to monitor the hemodynamic change and analyze the artery blood gas (Bayer 368, Germany). After hepatizing with 500 IU/kg heparin, the left femoral artery, and right internal jugular vein were cannulated to construct the CPB circuit. Moreover, the CPB circuit also contained a venous reservoir, roller pump, and membrane oxygenator. The CPB circuit was primed with 11 ml hydroxyethyl starch solution, which contained 0.2 mL heparin and 0.5 ml 7% sodium bicarbonate solution. During CPB, the flow rate CPB was gradually increased to 100 ml/kg/min and maintained for 60 min [14] under normothermic conditions. The CPB was administrated for 60 min. After CPB, the rats in sham and CPB groups were injected with saline (0.5 ml), and rats in Ac and Ac/AKT1 groups were injected with Ac2-26 (1 mg/kg) [11]. During CPB, the mechanical ventilation was withdrawn and the isoflurane was delivered using Drager Vapor, which connected within the inspiratory gas-membrane oxygenator circuit. The arterial blood pressure was maintained between 50-60 mmHg with epinephrine continuous infusion.

After 60 min of CPB, the mechanical ventilation was returned and the CPB was withdrawn. The protamine (0.1 mg per 100 IU heparin) was injected to anti-heparinize and the 2000 U/kg penicillin was injected to anti-infect. After saturation of incisions, the rats were extubated when they recovered spontaneous breath. All the rats were sacrificed after 12 h of CPB.

### Liver function estimation

After 12 h, all the rats were sacrificed with overdosage of anesthetic. Peripheral blood was collected before CPB, and 12 h after CPB. The peripheral blood was centrifuged at 5000 g for 10 min and the serum was stored at -70 °C. The serum was used to analyze the concentration of aspartate aminotransferase (AST), alanine aminotransferase (ALT), and lactate dehydrogenase (LDH).

### Inflammation

The TNF-α, IL-1β, IL-10, and elastase in liver tissue and serum were detected before CPB, and 12 h after CPB with Elisa Kits (Boster, Wuhan, China).

### Oxidative stress response

Part of liver tissue was homogenized to test the protein concentration and oxidative stress response. The concentration and activity of Xanthine oxidase (XO), myeloperoxidase (MPO), Superoxide dismutase (SOD), synthesis of glutathione (GSH) and malondialdehyde (MDA) in liver tissue were detected.

### Liver injury

Liver tissue preserved in 10% formalin was embedded in paraffin and cut into 4 μm section. Liver sections were stained with hematoxylin and eosin and examined using a light microscope. The histological injury was evaluated by two blinded to this study. The liver histological was scared by analyzing the infiltration of inflammatory cells, hepatocyte necrosis, ballooning degeneration, and hyaline degeneration as follows [16]: Score 0, no visible cell damage. Score 1, focal hepatocyte damage on less than 25% of the tissue, seldom neutrophil infiltration. Score 2, focal hepatocyte damage on 25-50% of the tissue, and moderate neutrophil infiltration. Score 3, extensive, but focal, hepatocyte lesions and infiltration of neutrophils. Score 4, global hepatocyte necrosis.

The part liver was weighed and dried at 80℃ for 24 h and the wet/dry weight ratio.

### Detection of apoptosis

The apoptosis of liver tissue was analyzed by the terminal deoxynucleotidyl transferase-mediated dUTP-biotin nick-end labeling (TUNEL) kit (Roche, Shanghai, China). Briefly, the liver sections were deparaffinized and rehydrated in graded alcohol. The protein in section was digested with proteinase K and incubated in terminal deoxyribonucleotidyl transferase enzyme at 37 °C for 2 h, and then immersed in antidigoxigenin peroxidase for 30 min at room temperature. After washing 3 times with PBS, the sections were stained with diaminobenzidine–hydrogen peroxidase and Mayer’s hematoxylin. The nuclei with brown were apoptosis. The pathologist examined ten random fields of each section and counted the apoptotic cells. The apoptosis index was calculated by the ratio of positive cells to total cells.

### Western blot analysis

Part of liver tissue stored in liquid nitrogen was homogenized and the protein was extracted. The protein concentration was tested by the Bradford assay. Equivalent protein was injected into the gel and then electrotransferred to the PVDF membrane. The PVDF membranes were incubated with the GSK3β, eNOS, Bax, Bcl-2, cleaved-caspase-3, and NF-κB antibodies (all from Santa Cruz Biotechnology, Inc. Santa Cruz, USA). Twenty-four hours after incubation, the membrane was further incubated with a second antibody to react with horseradish peroxidase, and then visualized with chemiluminescence.

### Statistical analysis

All the data was analyzed using the GraphPad Pris 8.0 and the normality data was presented as mean (SD). The difference of all the data was analyzed by one-way analysis of variance, and the difference between the two groups was corrected with the Bonferroni. A *p*-value of < 0.05 was considered statistically significant.

## Results

### The effect of shRNA on the expression of AKT1

**Fig. 1:**
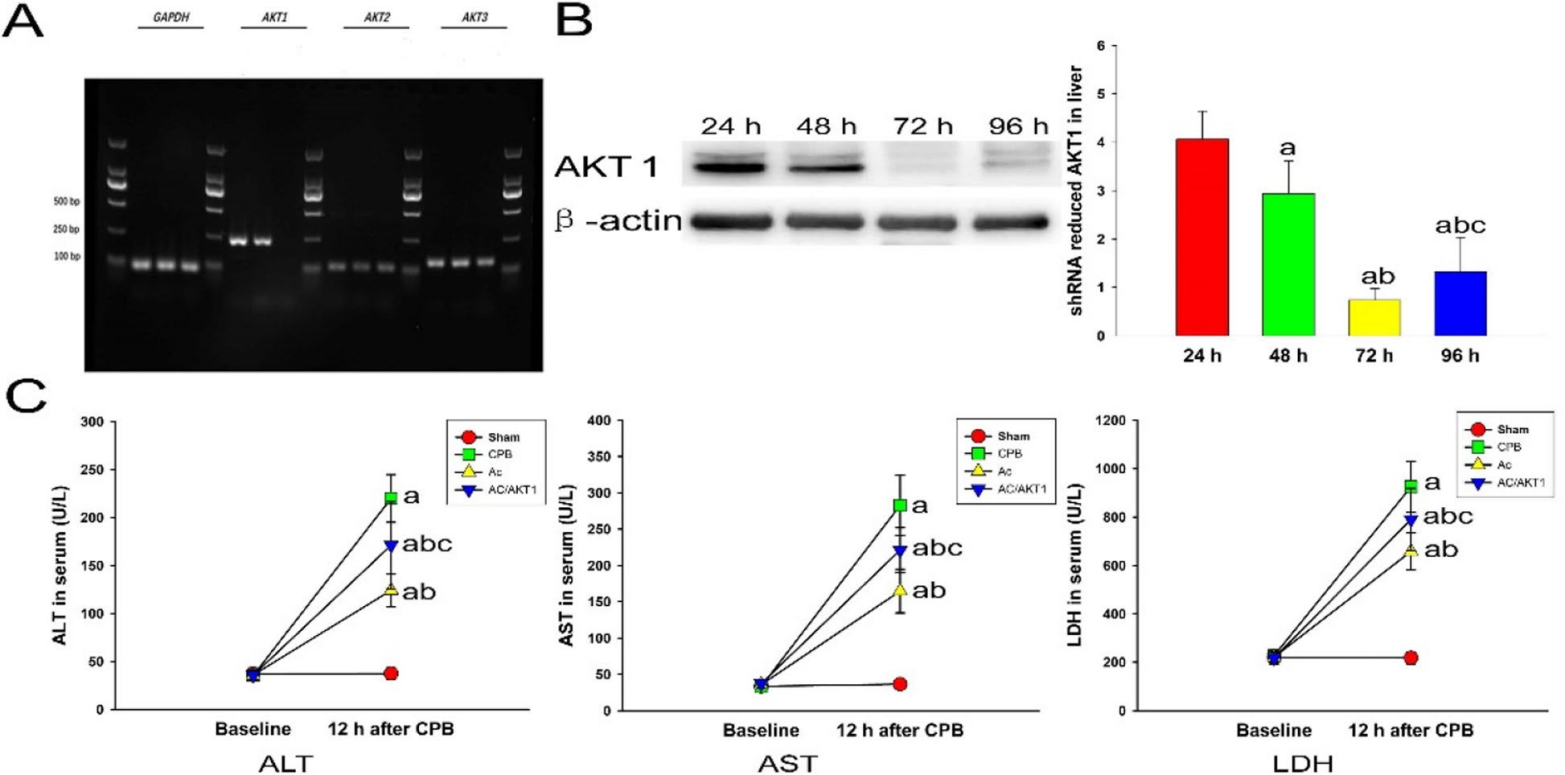
Effect of shRNA on AKT1 and Ac2-26 on liver function after CPB. The rats were injected with the shRNA to inhibit the expression of AKT1. The rats were sacrificed at 24, 48, 72, and 96 h after injection of shRNA. The livers were collected to detect the expression of AKT1 using Western blot. The results suggested that the peak effect of shRNA was at 72 h after injection (**A**). (a, *P* < 0.05, compared with 24 h timepoint; b, *P* < 0.05, compared with 48 h timepoint; c, *P* < 0.05, compared with 72 h timepoint) (  , 24 h timepoint;  , 48 h timepoint  , 72 h timepoint;  , 96 h timepoint). After 12 h of CPB, the ALT, AST, and LDH were significantly increased. Compared with the CPB group, the Ac2-26 notably decreased the ALT, AST, and LDH. Compared with the Ac group, the AKT reference RNA partly attenuated the protection of Ac2-26 on liver function. Mean levels of hepatic enzymes in four groups were significantly different. (a, *P* < 0.05, compared with the sham group; b, *P* < 0.05, compared with the CPB group; c, *P* < 0.05, compared with the Ac group) (  , Sham group;  , CPB group;  , Ac group;  , Ac/AKT1 group)

The shRNA only decreased the RNA expression of AKT1, but did not influence the expression of AKT2 and AKT3 (Fig. 1A). To manifest the efficacy of shRNA, we injected the shRNA into normal rats to observe the inhibited effect of shRNA on AKT1 in liver tissue. The expression of AKT1 in the liver was notably reduced by shRNA, especially for 72 h (Fig. 1B).

### Ac2-26 improved liver function

After CPB, the AST, ALT, and LDH were significantly increased compared with the sham group (*P* < 0.05). Compared with the CPB group, the AST, ALT, and LDH were decreased by the Ac2-26. The protection of Ac2-26 on liver function was partly reversed by the AKT1 interference (Fig. 1C).

### Ac2-26 attenuated the systemic and local inflammation

**Fig. 2:**
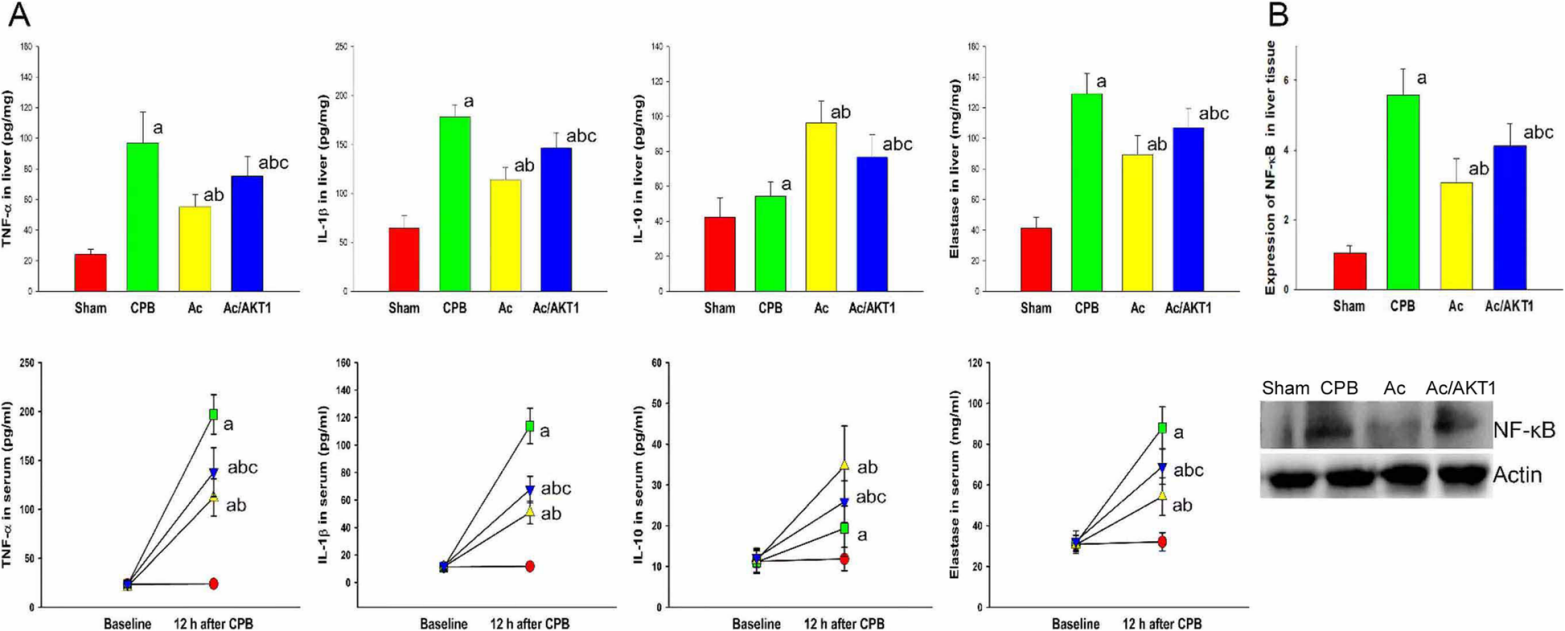
Effect of Ac2-26 on local and systemic inflammation after CPB. After CPB, the concentrations of TNF-α, IL-1β, IL-10, and elastase were significantly up-regulated. Compared with the CPB group, the Ac2-26 significantly downregulated the TNF-α, IL-1β, and elastase, but increased the IL-10 in liver tissue and serum. However, the regulation of Ac2-26 on cytokines was partly reversed by the AKT interference RNA (**A**). Moreover, we also detected the expression of NF-κB in liver tissue after CPB. The expression of NF-κB was increased by CPB, but decreased by Ac2-26. The AKT1 interference RNA notably attenuated the effect of Ac2-26 (**B**). (a, *P* < 0.05, compared with the sham group; b, *P* < 0.05, compared with the CPB group; c, *P* < 0.05, compared with the Ac group) (  , Sham group;  , CPB group;  , Ac group;  , Ac/AKT1 group)

The concentrations of TNF-α, IL-1β, IL-10, and neutrophils elastase were increased in rats who received CPB. After 12 h of CPB, the Ac2-26 significantly reduced the TNF-α and neutrophils elastase, but increased the IL-10 concentrations in serum (*P* < 0.05). Moreover, we also found that the expression of NF-κB was reduced by Ac2-26, but the reduction of NF-κB by Ac2-26 was lessened by AKT1 interference RNA (Fig. 2).

### Ac2-26 inhibited the oxidative stress response

**Fig. 3:**
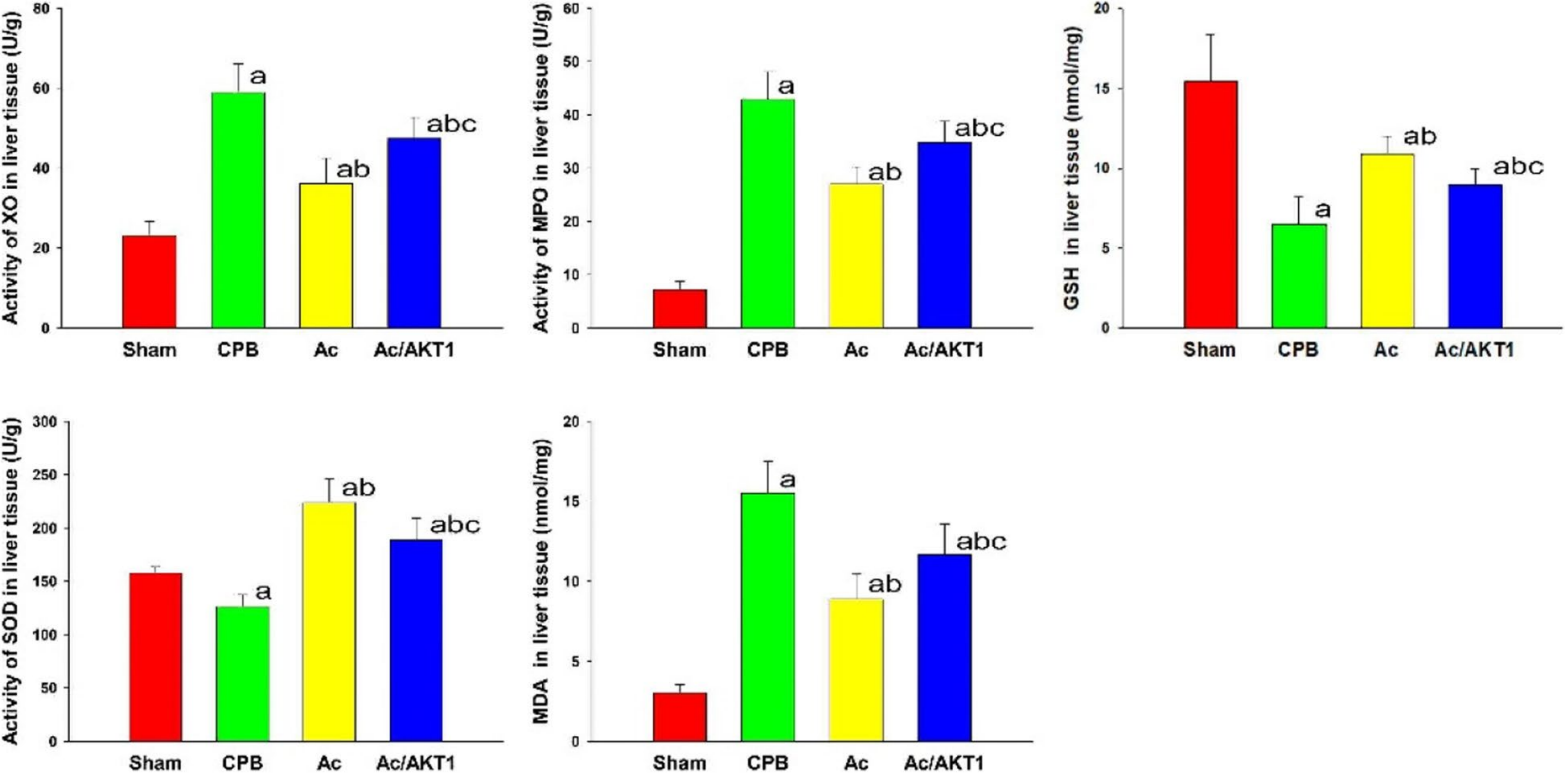
Effect of Ac2-26 on oxidative stress response in the liver after CPB. The activity of XO, MPO, SOD, and levels of GSH and MDA in the liver were tested. The activity of XO, MPO, and MDA levels were significantly increased, but the SOD and GSH were decreased. These results suggested CPB stimulated serious oxidative stress response. The Ac2-26 down-regulated XO, MPO, and MDA, but up-regulated the SOD and GSH, and this effect of Ac2-26 was significantly reduced by the AKT1 interference RNA. (a, *P* < 0.05, compared with the sham group; b, *P* < 0.05, compared with the CPB group; c, *P* < 0.05, compared with the Ac group) (  , Sham group;  , CPB group;  , Ac group;  , Ac/AKT1 group)

After CPB, the activity of XO, SOD, MPO, GSH, and concentration of MDA were significantly up-regulated, compared to the baseline and sham group (*P* < 0.05). Compared with the CPB group, the Ac2-26 significantly reduced the activity of XO, MPO, and MDA levels, but increased the activity of GSH and SOD levels. The regulation of Ac2-26 on oxidative stress response was partly lessened by the shRNA (*P* < 0.05) (Fig. 3).

### Ac2-26 ameliorated the liver injury

**Fig. 4:**
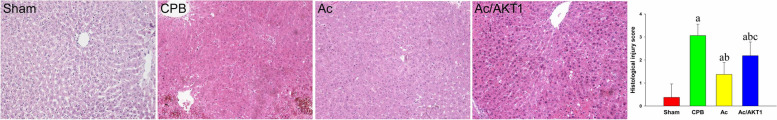
Effect of Ac2-26 on liver histological injury after CPB. The image of the sham group showed normal histological liver tissue. Compared with the sham group, the CPB led to severe liver injury, including prominently swollen, neutrophil infiltrations, and even hemorrhage. Compared with the CPB group, the Ac2-26 notably improved liver injury, but the improvement was partly lessened by the AKT1 interference RNA (X200). (a, *P* < 0.05, compared with sham group; b, *P* < 0.05, compared with CPB group; c, *P* < 0.05, compared with Ac group) (  , Sham group;  , CPB group;  , Ac group;  , Ac/AKT1 group)

There was no histological damage in the sham group. After activation of CPB, obviously histological damage was observed, including hepatic lobular necrosis, hyperemia in liver tissue, vacuolar degeneration of hepatocytes, neutrophil infiltration, derangement, and necrosis of the hepatocyte cord. Compared with the CPB group, the histological injury was attenuated by the Ac2-26. The score of liver injury was significantly lower in the Ac group compared to the CPB group. However, the protection of Ac2-26 on liver injury was partly inhibited by the AKT1 interference RNA (Fig. 4).

### Ac2-26 reduced the apoptosis

**Fig. 5:**
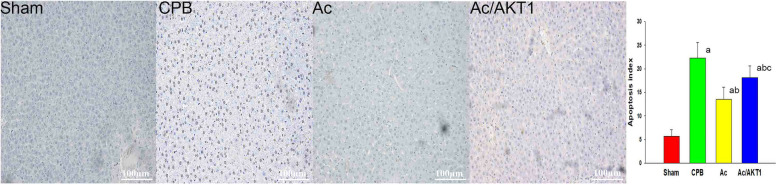
Effect of Ac2-26 on liver apoptosis after CPB. Apoptosis of the liver was estimated by TUNEL staining. CPB results in lots of liver cell apoptosis. Compared with the CPB group, the apoptosis was alleviated by Ac2-26, but this effect was reversed by AKT1 interference RNA. (a, *P* < 0.05, compared with the sham group; b, *P* < 0.05, compared with the CPB group; c, *P* < 0.05, compared with the Ac group) (  , Sham group;  , CPB group;  , Ac group;  , Ac/AKT1 group)

There were seldom apoptotic cells found in the sham group. Compared with the sham group, a lot of apoptotic hepatocytes were observed in rats induced by CPB (*P* < 0.05). Hepatocyte apoptosis induced by CPB was significantly inhibited by the Ac2-6. However, compared to the Ac group, the reduction of Ac2-26 on apoptosis was partially inhibited by AKT1 interference RNA (*P* < 0.05) (Fig. 5).

The apoptotic-regulated protein in liver tissue was detected. The Ac2-26 significantly reduced the expression of Bax and cleaved caspase-3, but increased the expression of Bcl-2. The modulation of Ac2-26 on apoptotic proteins was reduced by the knock-down of AKT1 (*P* < 0.05).

**Fig. 6:**
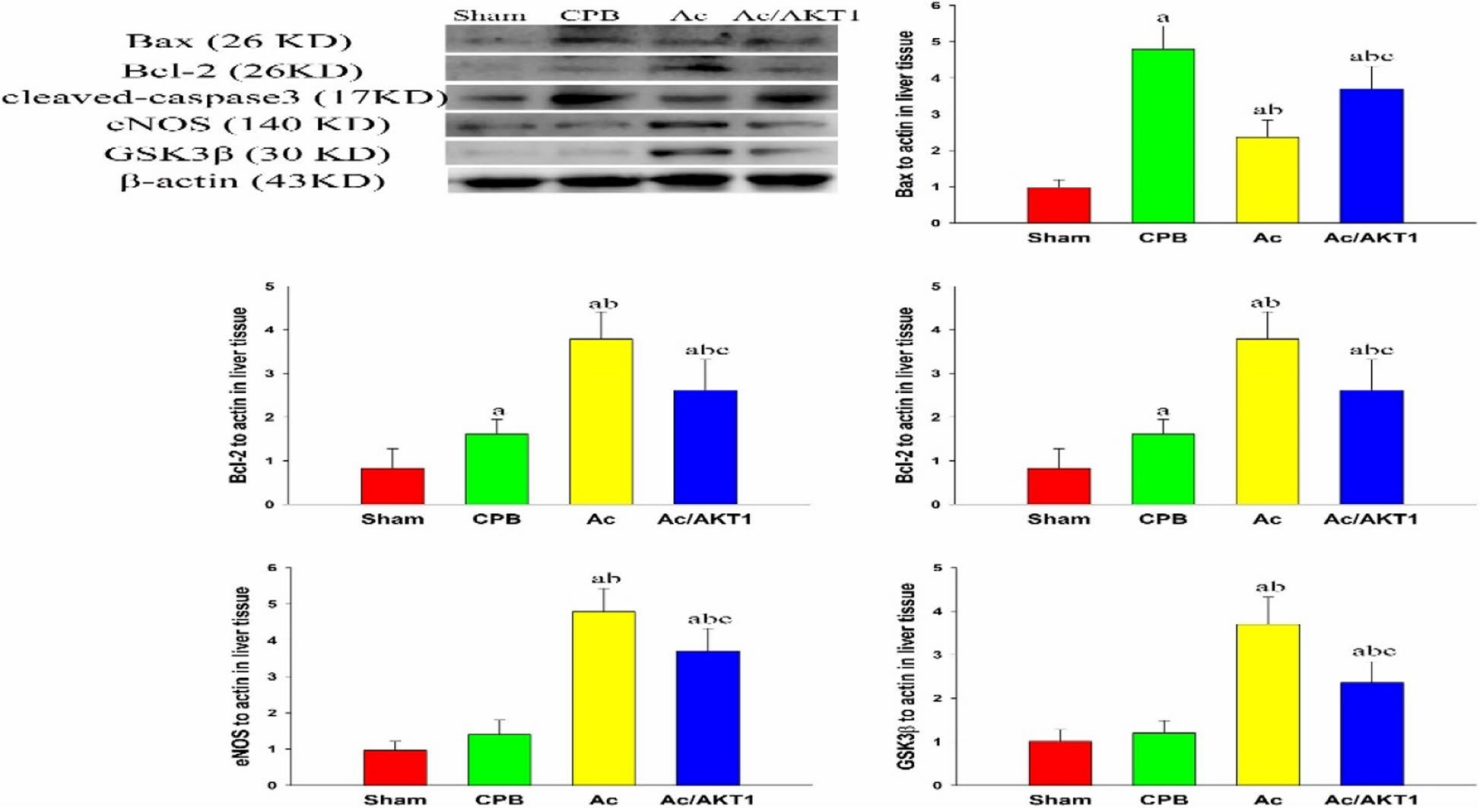
Effect of Ac2-26 on apoptotic protein after CPB. The pro-apoptotic protein Bax and cleaved caspase-3 were down-regulated, but the anti-apoptotic protein Bcl-2 was up-regulated by the Ac2-26. The regulation of Ac2-26 on apoptotic protein was attenuated by the AKT1 interference RNA. (a, *P* < 0.05, compared with the sham group; b, *P* < 0.05, compared with the CPB group; c, *P* < 0.05, compared with the Ac group) (  , Sham group;  , CPB group;  , Ac group;  , Ac/AKT1 group)

We also analyzed the eNOS and GSK3β in liver tissue. After CPB, the eNOS and GSK3β were slightly increased, but significantly up-regulated by the Ac2-26, but down-regulated by the shRNA (*P* < 0.05) (Fig. 6).

## Discussion

In this study, we found that Ac2-26 significantly improved liver function after CPB via anti-inflammation and anti-oxidative properties. The protection of Ac2-26 is mainly associated with AKT1.

The CPB is essential for the patients who undergo the cardiac surgery. However, exposure of blood to the circuit and liver ischemia/reperfusion will lead to severe SIRS and oxidative stress response. The inflammatory factors and reactive oxygen species (ROS) result in liver injury, which can influence the outcome of patients with cardiac disease [17]. We found that Ac2-26 significantly ameliorates lung injury after lung transplantation and CPB [11, 18]. Considering the crucial role of inflammation and oxidative stress response in liver injury after CPB, we speculated that Ac2-26 can reduce liver injury after CPB. Previous studies indicated that the liver injury peaked at 3 h after CPB and gradually decreased, but still nearly 5 times than baseline [2]. Firstly, we evaluated the effect of Ac2-26 on liver function and liver injury. In the rats that received CPB, the ALT, AST, and LDH were significantly increased, which agrees with previous studies [2]. The increase of ALT, AST, and LDH indicated severe hepatic dysfunction and hepatocyte injury after CPB. The higher liver enzymes in this study were different from clinical work. The major reason may be that the rats in this study did not receive additional medical drugs and treatments to inhibit the local and systemic inflammation. Moreover, the blood pressure in this study was maintained within 50-60 mmHg, this lower blood pressure might result in low perfusion in the liver and further lead to liver ischemia/reperfusion injury. Compared with the CPB group, the ALT, AST, and LDH were significantly reduced by the Ac2-26. These results suggested that Ac2-26 improved the liver function. Moreover, the liver histological injury and apoptosis also indicated that Ac2-26 reduced the liver injury induced by CPB.

During CPB, the inflammatory cells such as neutrophils and macrophages were activated and recruited into the organ which caused the injury. After infiltration, these inflammatory cells will produce various cytokines and lead to organ injury. In this study, the results of histological and cytokines in the liver and serum indicated that there were lots of neutrophils and macrophages infiltrated in liver tissue. Moreover, the activated inflammatory cells also secreted the cytokines and aggravated the SIRS. As we know, the TNF-α and IL-1β had significantly increased after CPB and were considered to be the indication of SIRS. The TNF-α and IL-1β played a pivotal role in liver injury in different liver injury models [2, 19, 20]. The TNF-α not only directly injured the hepatocyte [21, 22], but also triggered the extrinsic apoptosis. The IL-1β also contributed the inflammation and induced liver injury [23, 24]. The elastase could damage the basement membrane of hepatocytes, and extracellular matrix components and inhibit the endothelial production of prostacyclin [25], and elastase inhibitors can ameliorate liver injury [26]. In this study, the Ac2-26 significantly reduced TNF-α, IL-1β, and neutrophils elastase. In addition, the Ac2-26 reduced the inflammation after CPB also associated with an increase of the anti-inflammatory factor IL-10. As an important anti-inflammatory factor, IL-10 can inhibit the synthesis of proinflammatory cytokines, including TNF-α, IL-1β, and IL-6 [27]. As an endogenous glucocorticoid-regulated anti-inflammatory [28], Ac2-26 has been indicated to prompt the expression of IL-10 to regulate inflammation [29, 30]. These results suggested that Ac2-26 ameliorated liver injury and protected liver function may be associated with anti-inflammation.

In addition to inflammation, the oxidative stress response also plays a key role in liver injury. During CPB, the blood flow of the liver is significantly reduced and the hepatocyte will cause ischemia and reperfusion injury [2, 31]. After CPB, the activity of XO and MPO increased and contributed to the production of ROS. The XO generated various ROS and these ROS take part in various liver injury [32, 33]. The activity of MPO not only is a marker of neutrophils, but also plays an important role in the production of ROS [34]. In addition, as the final product of oxidative stress response, the MDA is usually the indication of the oxidative stress response [35]. In contrast, SOD and GSH are known as the main anti-oxidase factors [18]. In this study, all the indicators were significantly increased after CPB. These results suggested that CPB induced a severe oxidative stress response. Also, we found that Ac2-26 significantly decreased the activity of XO and MPO, and reduced the levels of MDA in liver tissue. In contrast, the Ac2-26 increased the activity of SOD and GSH after CPB. Therefore, we concluded that Ac2-26 can inhibit the oxidative stress response. These results were consistent with previous studies [9, 11].

Moreover, in this study, we also investigated the effect of Ac2-26 on the apoptosis of liver cells after CPB. As we know both the ROS and inflammatory cytokines result in apoptosis, and the apoptotic cells will influence the liver function and outcome of patients [36, 37]. In this study, we found the apoptotic liver cells were significantly increased after CPB, and the apoptosis was significantly lessened by the Ac2-26. The anti-apoptotic effect of Ac2-26 may be associated with the inhibition of Ac2-26 on oxidative and local inflammation induced by CPB.

In our previous study, we found the protection of Ac2-26 on lung injury mainly depended on the promotion of eNOS [11], and the production of eNOS mainly depended on the AKT1 [38]. Moreover, the GSK3β, which is important down-stream of AKT1, exerts survival, proliferation, and growth effect [39, 40], and activation of AKT/GSK3β/eNOS pathway has been indicated to present the anti-inflammation and apoptosis effect [41–43]. Therefore, to investigate the mechanism of Ac2-26 on liver injury after CPB, we used the rat knockdown AKT1 by shRNA to perform the CPB model, and injected the Ac2-26. In this study, all the protection of Ac2-26 was reversed in rats that received shRNA. These results indicated that the protection of Ac2-26 on liver injury after CPB mainly depended on the AKT1/GSK3β/eNOS pathway.

### Limitation

There were some limitations in this study. First, we did not observe the long-term effect of Ac2-26 on liver injury after CPB, because other articles and my preliminary trial suggested the inflammation peaked at 8-12 h after CPB and the liver function slowly recovered as time went on. Second, we only investigated the effect of AKT1 on the protection of Ac2-26, but not the deep protein pathway. This should be further investigated. Third, we did not deeply examine the effect of Ac2-26 on inflammatory cells. In our future study, we will culture and activate the inflammatory cells and survey the possible mechanism of Ac2-26 on inflammatory cells.

## Conclusion

According to the results, we concluded that Ac2-26 protected the acute liver injury, and improved the liver function via inhibition of inflammation, oxidative stress response, and apoptosis. The protection of Ac2-26 mainly depended on the promotion of AKT1 protein.

## Data Availability

No datasets were generated or analysed during the current study.

## Abbreviations

CPB: Cardiopulmonary bypass
shRNAs: Short hairpin RNA
SIRS: Systemic inflammatory response syndrome
ABG: Artery blood gas
AST: Aspartate aminotransferase
ALT: Alanine aminotransferase
LDH: Lactate dehydrogenase
XO: Xanthine oxidase
MPO: Myeloperoxidase
SOD: Superoxide dismutase
GSH: Synthesis of glutathione
MDA: Malondialdehyde
TUNEL: DUTP-biotin nick-end labeling
ROS: Reactive oxygen species
